# Treatment of osteonecrosis of the femoral head with focal anatomic-resurfacing implantation (HemiCAP): preliminary results of an alternative option

**DOI:** 10.1186/s13018-015-0199-3

**Published:** 2015-04-28

**Authors:** Onur Bilge, Mahmut Nedim Doral, Mustafa Yel, Nazim Karalezli, Anthony Miniaci

**Affiliations:** Department of Orthopaedics and Traumatology, Konya Necmettin Erbakan University, Meram Faculty of Medicine, Meram, 42080 Konya Turkey; Department of Orthopaedics and Traumatology, Hacettepe University, Faculty of Medicine, 06230 Ankara, Turkey; Cleveland Clinic Sports Health Center, 5555 Transportation Blvd, Garfield Heights, Ohio 44125 USA

**Keywords:** Femoral head, Osteonecrosis, Hip, Focal anatomic-resurfacing, Implantation

## Abstract

**Background:**

The optimal treatment of osteonecrosis of the femoral head has not been established yet. The aim of this study was to report preliminary clinical results of focal anatomic-resurfacing implantation for the treatment of osteonecrosis of the femoral head.

**Methods:**

Five patients (four male, one female) with seven surgical procedures, ages between 37 and 52 with an average age of 45.2 (+/− 7.2), diagnosed as femoral head avascular necrosis and who were unresponsive to conservative management or had failed previous surgical treatments were treated with a focal anatomic femoral head resurfacing between the years 2011–2012 and were retrospectively reviewed. Five patients with at least two years of follow-up, one left hip, two right hips, and two patients with bilateral hip surgery were included in this review. After safe surgical dislocation of the hip, full exposure of the femoral head was established. A focal-resurfacing implant matching patient anatomy and femoral head curvature was performed accordingly. Neither intraoperative or postoperative complications nor revision ensued. Visual analogue scores and Harris Hip Scores were recorded both preoperatively and at postoperative 2 years for all seven surgeries.

**Results:**

The mean follow-up period was 26.6 +/− 3.8 months, with a range between 24–33 months. The mean visual analogue scores were 8.9 +/− 0.9 preoperatively and 2.3 +/− 1.0 postoperatively at year two (*p* = 0.017). Harris Hip Scores at postoperative follow-up were found to improve significantly from good to excellent scores (86.0 +/− 7.9), compared with preoperative poor scores (26.7 +/− 11.8) (*p* = 0.018). The clinical improvements in visual analogue scores (VAS) and Harris Hip Scores were also found to correlate with each other (*p* < 0.05).

**Conclusions:**

In the present study, the alternative technique of focal anatomic hip resurfacing with HemiCAP® yielded preliminary successful results for the treatment of osteonecrosis of the femoral head. To the best of our knowledge, this is the first case series in the literature, reporting functional clinical results with the use of a focal anatomic-resurfacing implant for the treatment of focal femoral head osteonecrosis.

## Background

Osteonecrosis (ON) of the femoral head is a painful, progressive, and potentially debilitating disease, which affects patients in their third to fifth decades of life [1-3]. A variety of risk factors have been identified in the literature including trauma, alcohol abuse, chronic corticosteroid use, and coagulation disorders [4]. Although ischemia, direct cellular toxicity and altered differentiation of mesenchymal stem cells were postulated mechanisms of pathogenesis, the exact etiology and pathogenesis are still not certain [3,5].

Early diagnosis before collapse occurs is important in order to prevent subsequent collapse and osteoarthritis. The diagnosis requires a high index of suspicion, especially in the earliest stages. Deep groin pain is the most common early symptom. Limitation of range of motion (ROM) (especially internal rotation) is mostly evident at later stages. In general, the diagnosis is made by radiography and magnetic resonance imaging (MRI). MRI seems to be the best diagnostic tool with the highest sensitivity and specificity [6,7]. Moreover, MRI is the mainstay of all classification systems to stage ON, which currently guide treatment decisions and have prognostic importance. The two most commonly used classification systems are Ficat & Arlet, Ficat and Steinberg University of Pennsylvania [8-11]. The extent of the necrotic portion of the femoral head, which was also found to be a prognostic factor for collapse, could be measured with modified Kerboul method [12].

The treatment has been mostly based on the stage, extent, location, and cause of the ON together with the age of the patient. Currently, there are two main approaches for treatment: non-operative and operative. Both aim to relieve pain, to improve function, and to prevent progression to some extent. A variety of modalities have been described for the non-operative management of hip osteonecrosis in the literature including non-weight bearing, non-steroidal anti-inflammatory drugs, extra-corporeal shock wave treatment, pulsed electromagnetic therapy, and hyperbaric oxygen [13-21]. Many of these conservative treatments may only have a role in the early pre-collapse stages of the disease, with questionable limited success.

Currently, surgical treatment of ON of the femoral head includes the following: percutaneous drilling, core decompression with or without bone grafting, biological additions (stem cells, platelet rich plasma etc.), vascularized bone grafting, tantalum rods, proximal femoral osteotomies, and hip arthroplasties [22-32]. Although it seems that the most promising results were obtained with total hip arthroplasty, the optimal treatment for ON of the femoral head has not yet been established especially in young and middle-aged people [3,4].

The aim of this study was to report preliminary clinical outcomes of focal resurfacing implantation for ON of the femoral head as an alternative option of joint-preserving surgery of the hip. To the best of our knowledge, this is the first case series regarding the use of an alternative focal anatomic-resurfacing implant (HemiCAP**®**) for ON of the femoral head.

## Methods

### Patients

**Figure 1:**
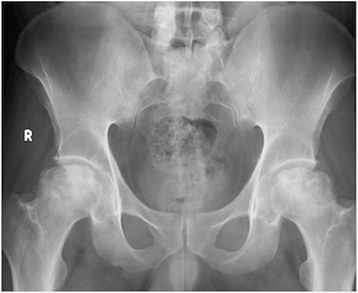
Preoperative X-ray. Anteroposterior pelvis X-ray demonstrating bilateral ON of the femoral head.

**Figure 2:**
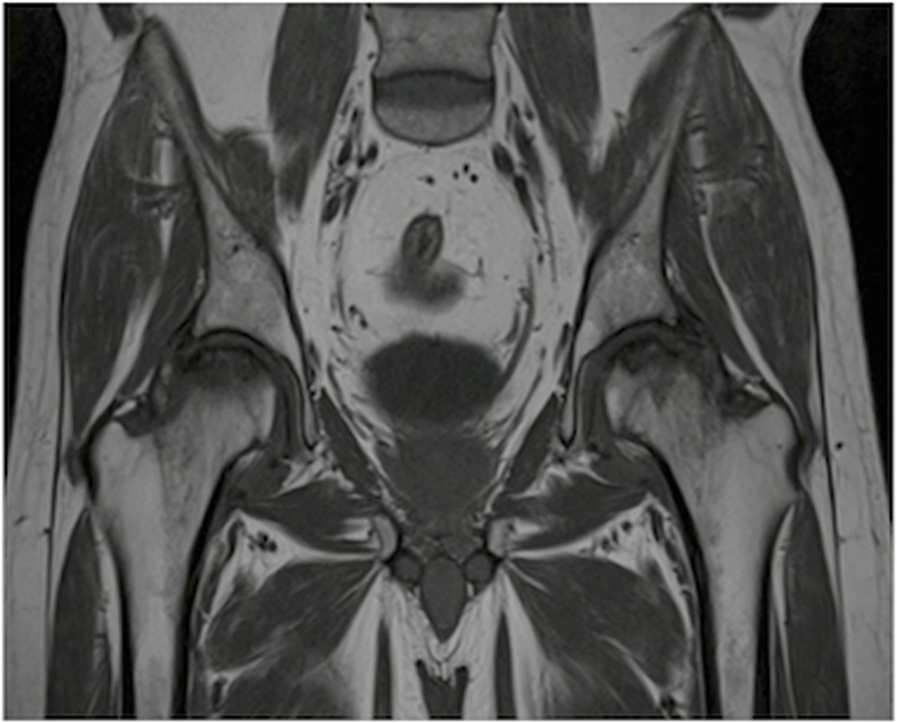
Preoperative MRI scan. T1-weighted coronal MRI scan demonstrating bilateral ON of the femoral head.

This study comprised a retrospective review of prospectively collected data for seven hips of five patients (four male, one female). The average age of the patients was 45.2 years (range 37–52 years). The demographic features of the patients are presented in Table 1. All patients presented with severe hip pain and limitation in hip ROM. The patients were diagnosed as Ficat-Arlet stage IIB, III, or IV femoral head avascular necrosis (two hips IIB, four hips III, one hip IV). Radiological imaging with X-ray and MRI of a patient with bilateral osteonecrosis of femoral head are shown in Figures 1 and 2. All the patients were unresponsive to non-surgical methods for at least one year. Two patients had previous core decompression, which eventually failed. After informed consent for surgery, patients were informed that the data would be reviewed for research. This study was also approved by the local Medical Ethics Committee.

**Table 1:** **Demographic features of patients**

**Patient number**	**Age**	**Sex**	**Previous managements**	**Risk factors**	**Surgical side**	**Ficat-Arlet stage**
1	48	Male	Conservative	Hodgkin’s Lymphoma	R	III
				Steroid use	L	III
2	51	Female	Conservative, core decompression	Breast cancer	R	IIB
				Radiotherapy		
				Steroid use		
3	37	Male	Conservative, core decompression	ITP	L	III
				Steroid use	R	IV
4	52	Male	Conservative, core decompression	Bronchial asthma	R	III
				Steroid use		
5	38	Male	Conservative	Alcohol abuse	R	IIB

*R:* Right, *L:* Left, *ITP:* Idiopathic Thrombocytopenic Purpura.

### Surgical technique

Informed consent was obtained from all patients discussing risks and complications including the possibility of conversion to total hip arthroplasty in the case of poor bone quality or fracture. None of the cases had to have a total hip arthroplasty. The seven surgeries were performed with the standard technique with a lateral approach and trochanteric flip osteotomy. Following perioperative prophylaxis with antibiotics and identification of the correct extremity, patients had spinal anesthesia and were placed in a lateral decubitus position. The surgical site was prepared and draped in a standardized sterile manner. Surgical dislocation of the hip was performed following a direct lateral approach to the hip and trochanteric flip osteotomy as described by Ganz et al. [33]. The hip was dislocated anteriorly with full vision of the femoral head and neck.

**Figure 3:**
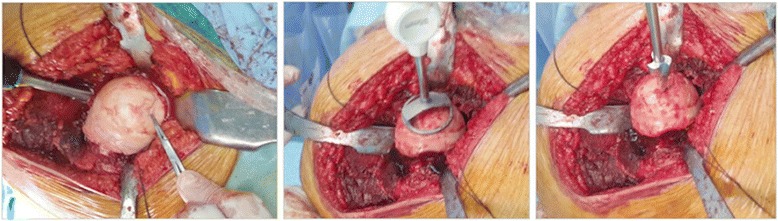
Surgical step 1. Following determination of the borders of the affected softened cartilage overlying osteonecrotic area and femoral head osteoplasty (if necessary due to impingement), a K-wire is passed through the sizing jig, into the center of the predetermined osteonecrotic area, which was debrided by using a power drill, with simultaneous lavage.

**Figure 4:**
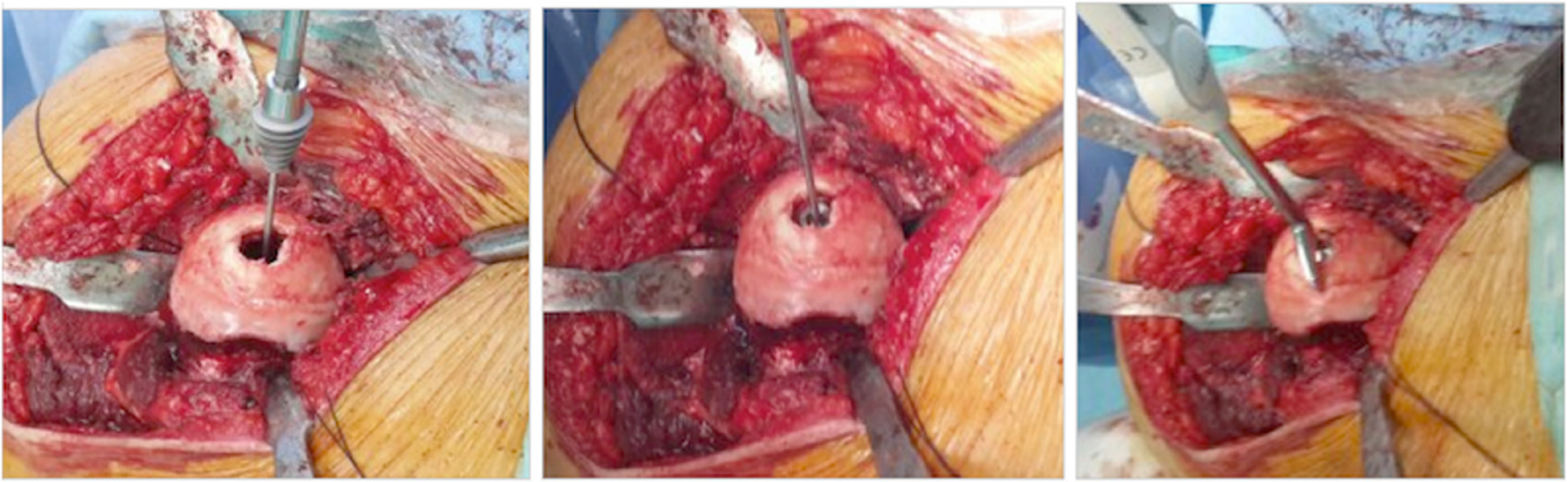
Surgical step 2. Implantation of the tapered titanium screw securely by the screwdriver, after proper taping over the guide K-wire and sizing of the medial, lateral, anterior, and posterior contours with the contact probe.

**Figure 5:**
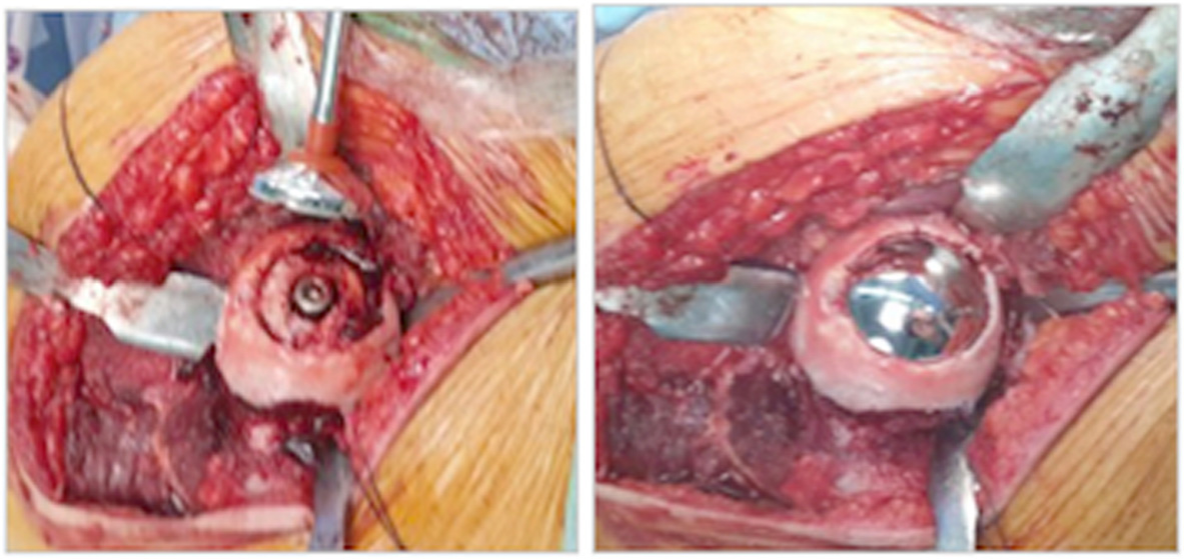
Surgical step 3. Placement of the anatomically fit sized, 35 mm diameter final implant with slight tapping with a mallet, after trialing and surface reaming. Final view of tapered interlocked CoCrMo articular-resurfacing component, approximately 0.5 mm below the peripheral healthy articular cartilage surface.

**Figure 6:**
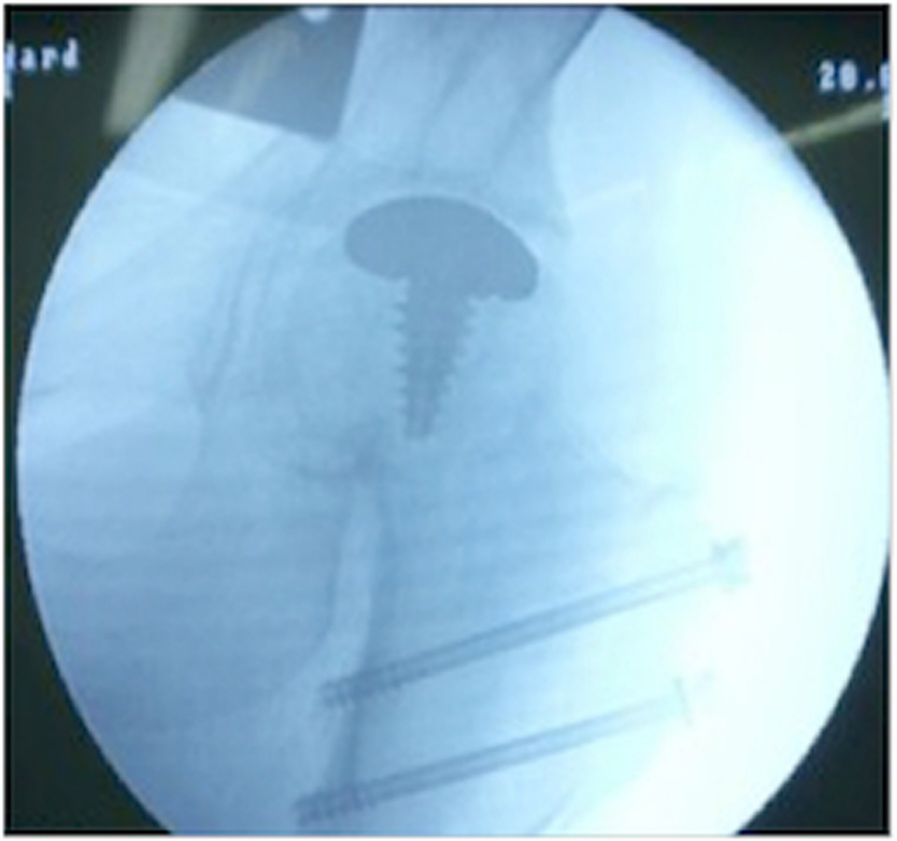
Intraoperative fluoroscopic view. Intraoperative fluoroscopic view showing a properly placed implant (HemiCAP®, Arthrosurface, Franklin, MA) with preservation of the joint space after reduction of the joint and final repair of the trochanteric osteotomy with two 4.5-mm cortical screws.

Firstly, the borders of the affected softened cartilage overlying the focal osteonecrotic area were determined. A femoral head osteoplasty was performed if necessary due to impingement. Then, a K-wire is passed through the sizing jig covering the entire lesion, into the center of the predetermined osteonecrotic area. After the debridement of this area by using a power drill over the guide K-wire, with simultaneous lavage, the tapered titanium screw was inserted securely by the screwdriver, following a proper taping over the guide K-wire. A contact probe was used in order to size the medial, lateral, anterior, and posterior contours correctly. There are seven different offset sizes available. After the final trial and surface reaming, an anatomically fit sized, 35 mm diameters, final implant was fixed on the pre-implanted titanium screw - approximately 0.5 mm below the peripheral healthy articular cartilage surface - with slight tapping with a mallet *via* an interlocking mechanism. The intraoperative fluoroscopic view was imperative in all cases to see the properly placed focal-resurfacing implant matching the patient’s anatomy and femoral head curvature (HemiCAP**®**, Arthrosurface, Franklin, MA) with preservation of the joint space after gentle reduction of the joint. Finally the trochanteric osteotomy was repaired with two 4.5-mm cortical screws. Neither perioperative nor intraoperative complications ensued in any patients (Figures 3, 4, 5, and 6).

It was imperative that good screw fixation was achieved for solid fixation of the implant. In each case, the screw was well fixed and often was difficult to turn into the underlying bone. In no instance was there any concern for fixation. We were prepared to utilize cement if necessary to augment fixation of the screw but this was not necessary.

### Postoperative management

**Figure 7:**
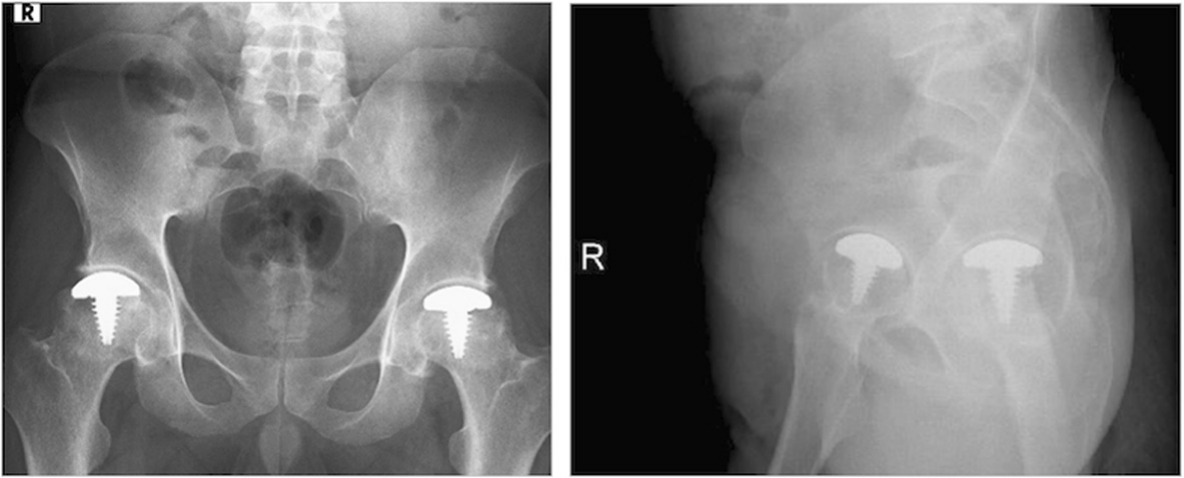
Postoperative X-rays. X-rays at postoperative second years of a 48-year-old male patient. These radiographs show the preservation of the joint spaces without loosening of the implants bilaterally. The screws were removed because of irritation in this patient.

**Figure 8:**
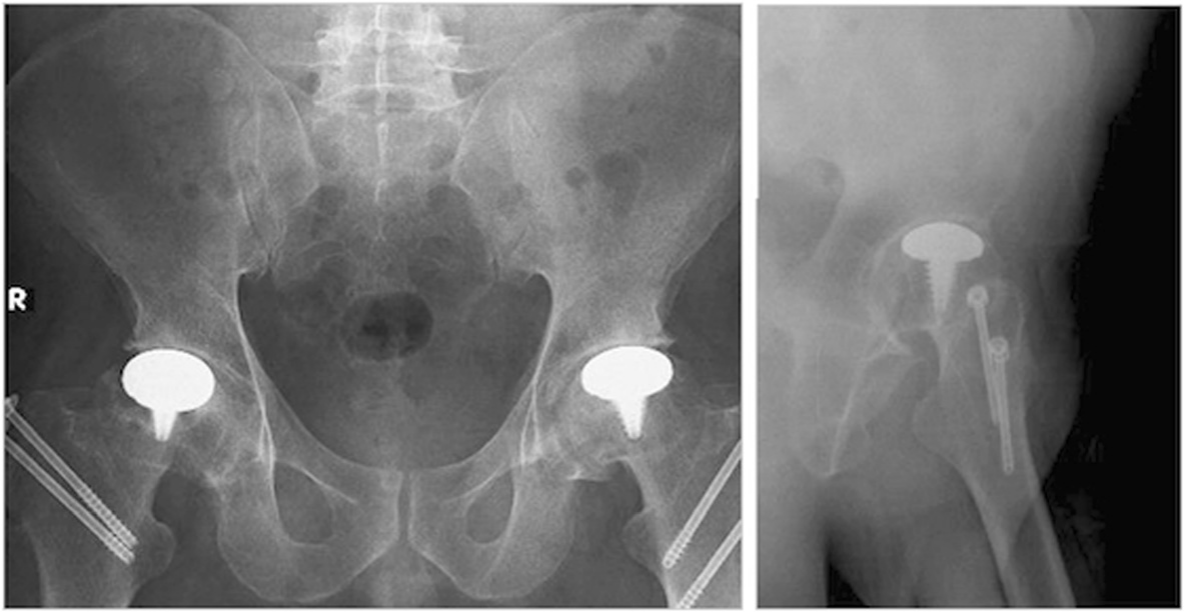
Postoperative X-rays. X-rays of a 37-year-old male patient at postoperative 24^th^ months.

Prophylactic antibiotic, which had been started perioperatively, was continued for 24 hours. Thromboprophylaxis with third generation low molecular weight heparin (Bemiparin, Hibor™) was started postoperatively at 12 hours and continued for 6 weeks. Neither early nor late postoperative complications ensued. The patients were mobilized with toe-touch weight bearing during the first four weeks, allowing the osteotomized greater trochanter to heal and sufficient implant-bone integration. Thereafter, the mobilization was progressed from partial to full-weight bearing. The osteotomies were healed at two months postoperatively. The screws were removed in two patients, as they became symptomatic. The patients returned back to their daily activities at a mean of three months postoperatively. At final radiological reviews, neither loosening nor subsidence of the implants were observed (Figures 7 and 8). Moreover, no progression of the osteonecrosis, acetabular reaction, head collapse, and progression to osteoarthritis were observed.

### Evaluation criteria

The clinical evaluation of the patients was done by using visual analogue score (VAS) and Harris Hip Score (HHS). These scores were recorded both preoperatively and at postoperative 2 years, for all seven surgeries of five patients. The data were retrospectively reviewed.

### Statistical methods

SPSS version 16.0 was used for statistics. Statistical analyses were performed using the Wilcoxon test and Spearman correlation test, for comparison between preoperative and postoperative values and for correlation of VAS and Harris Hip scores, respectively. The statistical significance was determined at *p* value <0.05.

## Results

**Figure 9:**
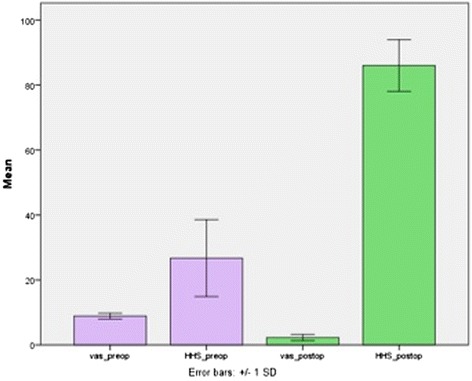
Mean VAS and Harris Hip Scores. Mean values (+/− SD, longitudinal lines) of preoperative (*purple bar*) and postoperative second year (*green bar*) VAS and Harris Hip Scores (VAS = visual analogue score, HHS = Harris Hip Score, preop. = preoperative, postop. = postoperative).

The mean follow-up period was 26.6 +/− 3.8 months (range 24–33 months). The mean VAS scores were 8.9 +/− 0.9 preoperatively and 2.3 +/− 1.0 postoperatively at year two (*p* = 0.017). Harris Hip Scores improved from 26.7 preoperatively to 86.0 postoperatively, which were in the good to excellent category (*p* = 0.018). The data were summarized in Table 2 and Figure 9. The preoperative mean scores for pain, functional status, and joint status were 13.6 +/− 5.6, 10.3 +/− 6.6, and 2.9 +/− 1.3, respectively. The respective values at postoperative 2^nd^ years were 45.0 +/− 5.2, 32.3 +/− 4.8, and 8.9 +/− 0.7. The lowest items having the poorest scores postoperatively were found to be the following: support, distance walked, and sitting. In addition, the improvements in VAS and Harris Hip Scores were found to correlate significantly with each other (*p* < 0.05).

**Table 2:** **Preoperative and postoperative 2** ^**nd**^ **year VAS and Harris Hip Scores**

**Patient number**	**Surgical side**	**Preoperative VAS**	**Postoperative VAS**	**Preoperative Harris Hip Score**	**Postoperative Harris Hip Score**
1	R	10	2	24	84
	L	8	1	51	94
2	R	8	2	22	88
3	R	10	3	15	87
	L	9	2	22	86
4	R	8	2	32	93
5	R	9	4	21	70
	Mean +/− SD	8.9 +/− 0.9	2.3 +/− 1.0	26.7 +/− 11.8	86.0 +/− 7.9
	*p values*		*0.017*		*0.018*

*VAS:* visual analogue score, *SD:* standard deviation.

## Discussion

The most important result of this study was that the use of the focal anatomic-resurfacing implant for the treatment of osteonecrosis of the femoral head yielded successful preliminary clinical results in seven hips of five patients at a minimum of two years following their surgical procedure. In addition, to the best of our knowledge, this study is the first case series related with the use of this alternative focal anatomic-resurfacing implant for this specific indication.

In general, the management of osteonecrosis of the femoral head consists of non-surgical and surgical methods. Non-surgical modalities such as non-weight bearing, pharmacological (bisphosphonates, anticoagulants, lipid-lowering agents, vasodilators), extra-corporeal shock wave treatment, pulsed electromagnetic therapy, and hyperbaric oxygen may have a role only in the early pre-collapse stages of the disease, with a questionable limited success [13-21].

Surgical methods can also be subdivided into two major groups: joint preserving options and total hip arthroplasty. Joint preserving options include the following: percutaneous drilling, core decompression with or without bone grafting, biological procedures (bone marrow stimulation or microfracture, mesenchymal stem cells), vascularized bone grafting, tantalum rods, proximal femoral osteotomies, and hip arthroplasties [22-32].

The most commonly performed surgical method is probably core decompression, which aims to decrease intraosseous pressure and to ameliorate the blood flow to the necrotic area and subsequent healing by creeping substitution. Despite these aims and its use at earlier stages of the disease process, the rate of further additional surgery requirements was reported as high as 80% [13,34,35]. The clinical results of this option had wide variations from 0% to 91% [36]. Moreover, Lee et al. found that intertrochanteric osseous pressure was significantly higher after multiple drilling and that these patients had poorer outcomes [37]. Interestingly, this result raises the question of whether the increase in pressure in the intertrochanteric region after core decompression increases the severity of the disease progression or not.

Another viable surgical option is free vascularized bone grafting, which has been advised for earlier stages, before collapse [3,35]. Although successful results were reported at a longer term [27,38,39], it is a highly technical and demanding procedure and is associated with significant donor site morbidities and progression of the osteonecrotic lesion.

When there is a collapse of the lesion, treatment becomes more difficult. Although it seems that the most promising results, especially at late stages, are reported with total hip arthroplasty, the optimal surgical treatment for ON of the femoral head has not been established, especially in the younger patients [3,4]. The first is the longevity of the implant and is a definite concern [40]. In addition, in contrast to its successful use in patients with primary coxarthrosis, THA was found to have a higher rate of failure in patients with osteoarthritis secondary to osteonecrosis [41,42]. Moreover, the results of THA were reported to be poor in the young age group, which these patients usually are [43-46]. In this context, the importance of hip-preserving surgery has also been stressed in the last decade recently [47]. Under the light of these findings, an alternative treatment option is required in this young age group. Although our study exhibited preliminary results at a mean follow-up of postoperative 26 months, the early results were promising, and we anticipate a better solution in young patients compared with THA, with the advantage of postponing the age for THA. As the number of selected younger patients - with focal ON of the femoral head - who will be treated with the hip HemiCAP® increase, the results will be easier to compare with those of THA.

Recently, short-term successful results were obtained with resurfacing arthroplasty in the treatment of osteonecrosis of the femoral head [48]. This procedure preserves bone stock in the femoral head, without compromising subsequent conversion to THA. It can also be used at postcollapse stages in young patients with good bone stock [3]. But, due to reported high revision rates to THA, mostly because of femoral neck fracture, and due to effects of metallic wear debris, most surgeons do not prefer resurfacing arthroplasty for the treatment of ON of the femoral head [49-51].

Compared with previously discussed alternative techniques, the hip HemiCAP® system has some advantages. At first, preoperative planning is not as detailed as total hip arthroplasty. It is a femoral head and joint-preserving surgery in which the patient’s own femoral head anatomy is protected to a maximal extent with little bony resection and can be used even in cases of collapse. After the entire exposure of the femoral head, removal of the necrotic area is followed by replacement of the defect by a two-part implant fixed in position with a large threaded screw. This implant matches each patient’s anatomy and femoral head contour. They are suitable for young and middle-aged patients with focal lesions. Another advantage is that in case of progression of osteoarthritis, or implant failure or fracture at any time, there is always a chance to revise to primary total hip arthroplasty.

Only four case reports were published in the literature to our knowledge of the use of HemiCAP® for femoral head pathologies. In the first study, Jäger et al. reported successful results in a 22-year-old female patient having traumatic osteonecrosis of the femoral head, with one-year follow-up [52]. In a second study, partial resurfacing was performed successfully following a subcapital femoral varus osteotomy in a 16-year-old male patient, with a two-year follow-up [53]. In a study of Mahmud et al., a 24-year-old male with osteochondral defect of the femoral head was successfully treated with HemiCAP®, with five-years follow-up [54]. Recently, a patient having an osteochondral lesion of the hip, who was treated with partial femoral resurfacing, has been reported with the longest follow-up of six years [55].

To our knowledge, the present study is the first report of a consecutive case series with the use of HemiCAP® for the treatment of focal ON of the femoral head. But, there are some limitations of this study, which should be discussed. Firstly, this study compromises a small number of patients, but this is not a common condition and large studies are difficult to do to collect enough cases. Another limitation is the retrospective evaluation of the prospectively collected data. A prospective, multi-center study evaluating this procedure over a long term would give more definitive results. However, the procedure seems to have some promise in the treatment of osteonecrosis with advanced stages and collapse or as a salvage of other failed procedures. The biggest advantage is that the anatomy is preserved so that future procedures are not compromised.

## Conclusions

As a result, the optimal and ideal treatment for the focal osteonecrosis of the femoral head has not been established, especially for young and middle-aged patients. Although THA is often used for these patients, implant longevity and subsequent revisions have still been problems in these age groups. To the best of our knowledge, this study is the first case series in the literature, reporting successful clinical results with the use of an alternative focal, anatomic, limited-resurfacing implant (HemiCAP**®**) for the femoral head and joint preserving treatment of focal osteonecrosis, and we would recommend it as a potential alternative treatment option for these patients.

## Abbreviations

ON: osteonecrosis
ROM: range of motion
MRI: magnetic resonance imaging
R: right
L: left
ITP: idiopathic thrombocytopenic purpura
VAS: visual analogue score
HHS: Harris Hip Score
SD: standard deviation
THA: total hip arthroplasty
